# Temperature Dependence of Reaction Kinetics in a Hybrid GaAs Solar–Fuel Cell Device

**DOI:** 10.1021/acs.jpclett.4c02018

**Published:** 2024-09-10

**Authors:** Mahdi Alizadeh, Shengyang Li, Seyed Ahmad Shahahmadi, Jani Oksanen

**Affiliations:** † Engineered Nanosystems Group, School of Science, Aalto University, Tietotie 1, 02150 Espoo, Finland; ‡ Department of Chemistry, China Agricultural University, Beijing 100193, China

## Abstract

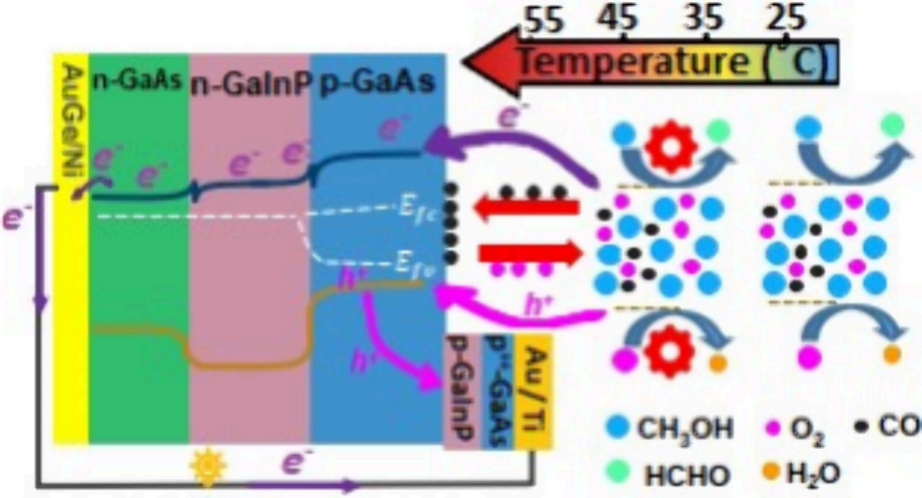

The recently proposed single-electrode fuel cell (SEFC) is based on the chemovoltaic effect in a semiconductor p–n junction and as a hybrid device also allows operation as a photovoltaic cell. This study investigates the temperature dependence of the chemovoltaic effect in GaAs/GaInP p–n double heterojunction SEFC devices in the presence of both liquid and vapor methanol as a fuel. The experimental results reveal that increasing the temperature from room temperature to around 45 °C significantly enhances the fuel cell’s performance by accelerating the electrochemical oxidation and reduction reactions injecting electrons and holes into the semiconductor bands. However, further increase in the fuel temperature, nearing the boiling point of methanol, leads to adverse effects on the cell’s performance when submerged in the liquid fuel but still shows moderate improvement when operating with the vapor-phase fuel. These results provide insight into the kinetics of the chemovoltaic effect in a hybrid solar–fuel cell device.

Since the advent of fuel cells (FCs) in the mid-19th century,^1^ these cells have primarily been designed with three essential components: a cathode, an anode, and an electrolyte. The fuel and oxidants are separately supplied to the anode and cathode, resulting in electrochemical reactions on the electrodes and electron/ion generation or consumption. The electrolyte, as a multifunctional component, enables ion movement between the two electrodes, inhibits gas permeation, and prevents electron conduction. This structure introduces specific requirements for the device geometry and especially for the ionic conductivity and mass transport, which further influence the polarization losses in the electrolyte and at the electrolyte–electrode interfaces.^2−4^ For instance, it has been reported that to reduce the resistive losses, the ionic conductivities of all electrolytes in fuel cells should exceed 10^–2^ S cm^–1^,^2^ potentially inducing complexity to the cell design.

Recently, we introduced an “electrolyte-free fuel cell” based on a GaAs diode, demonstrating electrochemical fuel oxidation and oxidant reduction reactions directly on its conduction and valence bands, respectively.^5^ The theoretical and experimental results demonstrated that redox reactions occurring between a fuel and an oxidizer on the cell’s surface can chemically excite the semiconductor diode, which can simultaneously also function as a solar cell. This excitation in turn triggers a splitting of the Fermi level for both the conduction and valence bands, thereby enabling the direct conversion of chemical energy into electricity through the so-called chemovoltaic effect in a single-electrode fuel cell (SEFC).

Ideally, harnessing the chemovoltaic effect could lead to a new generation of FCs that can convert both chemical and optical energy to electricity, but the fundamental properties of such chemovoltaic FCs are still largely unknown, particularly regarding reaction kinetics, catalysts, surface stability, and their dependence on external conditions. Furthermore, a deeper comprehension of the energy conversion process and its potential requires further efforts in system optimization and improvements in the cell design. Temperature is one of the key parameters that significantly affects the reaction kinetics, especially in methanol-based fuel cell devices.^6−8^ The dependence of the reaction rate on temperature can be characterized using the activation energy, which represents the energy barrier that reactant molecules must overcome for the reaction to proceed. In this work, we investigated the effect of temperature on SEFC performance by exposing GaAs-based double heterojunction (DHJ) test devices to both liquid- and vapor-phase fuels. Increasing the temperature increases both the open-circuit voltage (*V*_oc_) and short-circuit current density (*J*_sc_) from 120 mV and 0.95 μA/cm^2^ to 260 mV and 2.8 μA/cm^2^ for the liquid phase and from 155 mV and 0.7 μA/cm^2^ to 350 mV and 3.5 μA/cm^2^ for the vapor phase, respectively. The observed temperature dependence suggests that even moderate changes in the temperature can significantly improve the cell performance, leading to, e.g., up to a 5-fold increase in induced current and a 2-fold increase in *V*_oc_, and provides more insight into the reaction kinetics.

In this work, GaAs/GaInP DHJs deposited by metal–organic vapor-phase epitaxy (MOVPE) were used as the electrodes. In contrast to the previously used single heterojunction devices,^5^ the DHJ structures have been observed to exhibit a lower leakage current, which improves device yield and facilitates the detection of the electrochemically generated current. In addition, growing the GaAs layer between the GaInP layers confines charge carriers more efficiently to the active region, enhancing charge transport within the device. The layers of the device structure are illustrated in Figure 1a,b, illustrating also the overall experimental setup. Our GaAs-based device was grown at a constant growth temperature of 595 °C (wafer surface temperature) and with V/III ratios of approximately 11 for GaAs and 65 for GaInP. The doping sources, including diethylzinc for p-type doping and disilane for n-type doping, were utilized during the growth process and calibrated by Hall effect measurements.

**Figure 1:**
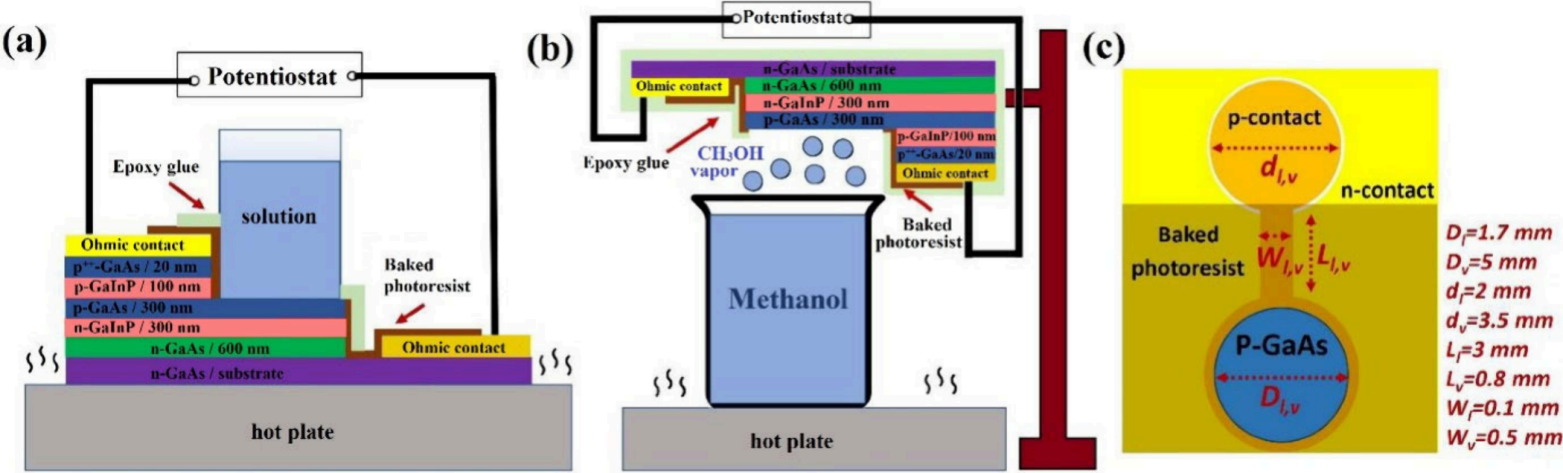
Schematic side view of the GaAs/GaInP chemovoltaic cell measurement setup for (a) liquid- and (b) vapor-phase fuels. In (b), the device size has been scaled up for better visualization. (c) Top view of the chemovoltaic device with corresponding dimensions for liquid (l) and vapor (v) phases.

After the growth, processing of the grown samples was conducted in a clean room where the device materials were prepared by maskless aligner photolithography and wet chemical etching. Nitric acid powder was dissolved in deionized water (weight ratio 1:1), and the prepared acid solution (HNO_3_(l)) was mixed with hydrogen peroxide and water (HNO_3_(l):H_2_O_2_:H_2_O volumetric ratio = 4:1:4) and was used as the etchant for GaAs etching. The GaInP layers were etched with 7 M hydrochloric acid. For both liquid- and vapor-phase measurements, the mesa shape was identical, consisting of two circles connected with a narrow bridge as shown in Figure 1c. Two different device sizes (0.06 cm^2^ and 0.30 cm^2^) were prepared for liquid and vapor experiments, respectively, with the larger devices allowing wires to be attached more easily on the contact pads using silver paste. One of the circles served as the active area, and the other one was covered by the contact metal. Au (100 nm)/Ti (30 nm) thin films were deposited on the topmost layer of the mesas, covering the contact pad, the outer rim of the active area, and the bridge between them. The area outside of the mesas was covered by a AuGe (110 nm)/Ni (10 nm) bilayer film as the n contact. After contact deposition, the devices were rapidly annealed at *T* = 350 °C for *t* = 15 s. Subsequently, another lithography step was used to selectively etch the GaAs cap and p-GaInP layers from the active area, exposing the p-GaAs/n-GaInP/n-GaAs structure with the p-GaAs surface for the electrochemical measurement. Finally, the processed devices underwent encapsulation using a photoresist heated at 200 °C for 2 h, leaving only the active area and contact pads open for the electrochemical measurement. As the fuel compartment for the liquid-phase measurements, a glass tube was affixed on the active area of the devices using an epoxy adhesive, whereas the vapor -phase measurement was performed through wires attached to the p and n contact pads using silver paste. The vapor-phase devices were additionally encapsulated with epoxy glue, leaving only the active area open for the measurement. To prepare the surfaces prior to the actual electrochemical measurements, we submerged the active areas of both types of devices in the 7 M HCl solution for 1 min.

The liquid-phase electrochemical measurements were conducted by placing the devices with the fuel compartment filled with CH_3_OH or CH_3_OH + 0.05 M H_2_O_2_ solution on a hot plate set at room temperature (RT), 35, 45, or 55 °C. For the vapor-phase measurement, a beaker filled with pure CH_3_OH was placed on the hot plate, and the fabricated device was clamped above the beaker with around a 1 cm distance to the liquid surface. The electrical characteristics of the devices were analyzed with conventional *I*–*V*, chronoamperometry, and chronopotentiometry measurements.

In our previous studies,^5,9^ both numerical simulations and experimental findings indicated that when a semiconductor is exposed to appropriate redox couples with their redox potentials straddling the quasi-Fermi levels of the semiconductor, the semiconductor can be chemically excited. In this study, we performed several experiments measuring the impact of temperature on the performance of the GaAs-based SEFC device. The chemovoltaic devices used in this work were GaAs/GaInP DHJs fabricated by MOVPE. Because of this particular structure, these devices are also capable of functioning as photovoltaic devices.

Figure 2a,b displays the logarithmic *J*–*V* results of the cells in the presence and absence of liquid methanol and dissolved oxygen or oxidizer in the fuel compartment pipe under complete darkness. The linear *J*–*V* plots are shown in Figure S1. As expected, in the absence of CH_3_OH or CH_3_OH + 0.05 M H_2_O_2_ solution, *V*_oc_ and *J*_sc_ of the cell are zero. The cell produced *V*_oc_ values of 120 and 150 mV and *J*_sc_ values of 0.95 and 1.2 μA/cm^2^ when the fuel compartment was filled with liquid CH_3_OH or CH_3_OH + 0.05 M H_2_O_2_, respectively, at RT. Figure 2c shows the *J*–*V* results of the cells exposed and unexposed to methanol vapor in the dark. Without exposure to the vapor fuel, both the *V*_oc_ and *J*_sc_ values are negligible. However, when the device is placed above a container filled with methanol at RT, both *V*_oc_ and *J*_sc_ increase to 155 mV and 0.7 μA/cm^2^, respectively. Figure 2a–c also depict the *J*–*V* results of the cells under various temperatures (*T*) ranging from 35 to 55 °C. In the absence of the solutions and vapor, increasing the temperature from RT to 55 °C results in *V*_oc_ and *J*_sc_ remaining at zero (see Figure S2). As can be seen in Figure 2d, which shows the dependence of *V*_oc_ and *J*_sc_ on the temperature, increasing the temperature to 45 °C increases both *V*_oc_ and *J*_sc_ of the devices exposed to liquid CH_3_OH to 215 mV and 2.1 μA/cm^2^, respectively. Further increasing the temperature to 55 °C reduces *V*_oc_ and *J*_sc_ to 95 mV and 0.1 μA/cm^2^, respectively. A similar trend is observed when H_2_O_2_ with a low concentration (0.05 M) is added to the solution, as displayed in Figure 2b,d: *V*_oc_ first increases to 260 mV as the system is heated to 45 °C and then decreases to 125 mV at *T* = 55 °C. Meanwhile, *J*_sc_ peaks at 2.8 μA/cm^2^ when the temperature is set at 45 °C and then drops to 0.2 μA/cm^2^ once the device is heated to 55 °C. For the vapor-phase devices, *V*_oc_ and *J*_sc_ keep increasing throughout the measured temperature range of 25–55 °C. The values increase to 350 mV and 3.5 μA/cm^2^, respectively, when the fuel is heated to 55 °C. However, despite large increases in *V*_oc_ (from 155 to 320 mV) and *J*_sc_ (from 0.7 to 2.5 μA/cm^2^) when the temperature was increased from 25 to 45 °C, only a modest rise was noted when the fuel temperature increased from 45 to 55 °C. In this experiment, however, the temperature of the cell itself was not controlled and may be lower than the temperature of the liquid.

**Figure 2:**
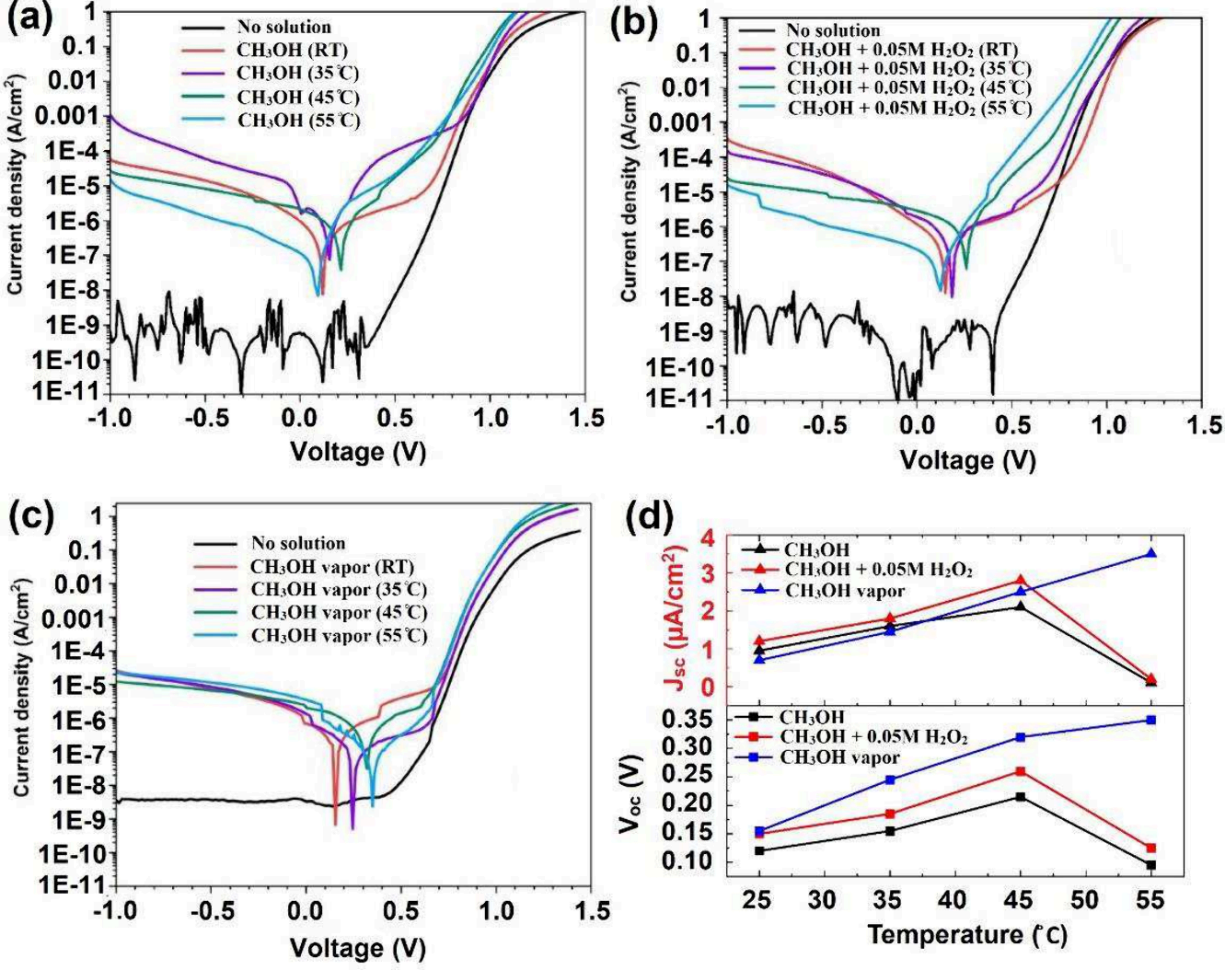
(a–c) *J*–*V* curves of the GaAs/GaInP cell in the dark at different temperatures with and without exposure to (a) CH_3_OH, (b) CH_3_OH + 0.05 M H_2_O_2_, and (c) CH_3_OH vapor. (d) *V*_oc_ and *J*_sc_ as functions of temperature for the chemovoltaic cells operated with CH_3_OH and CH_3_OH + 0.05 M H_2_O_2_ solutions and CH_3_OH vapor.

Chronopotentiometry (at a fixed current of 0 A) and chronoamperometry (at a fixed voltage of 0 V) measurements were carried out to evaluate the durability of the device for electricity generation, and the results are presented in Figure 3. Figure 3a–c shows an approximately constant electrical energy production within a 30 min measurement time for CH_3_OH (Figure 3a) and CH_3_OH + H_2_O_2_ (Figure 3b) in the temperature range of 25–45 °C and for CH_3_OH vapor (Figure 3c) in the temperature range of 25–55 °C. Furthermore, Figure 3a,b shows rapid decay in *V*_oc_ of the cell operated with CH_3_OH solutions at *T* = 55 °C, as *V*_oc_ decreases from 150 to 20 mV over the 30 min period, potentially due to the reduced solubility of oxygen near the boiling point. The *V*_oc_ decay is less pronounced in the mixture of CH_3_OH and H_2_O_2_ with a low concentration (0.05 M), as the value decreases by 40% from its maximum of 170 mV at the beginning of the measurement. Since the H_2_O_2_ concentration in the CH_3_OH + H_2_O_2_ system is low (0.05 M), it is likely that the amount of dissolved oxygen still plays a significant role in reducing the performance near the boiling point. Figure 3d–f illustrates the chronoamperometry results of the chemovoltaic cell at a fixed voltage of zero in the presence of both liquid- and vapor-phase fuels. The observed trends in the chronoamperometry results are similar to the chronopotentiometry ones. The current–time results of our SEFC device, operating with liquid fuels at the highest temperature (*T* = 55 °C), exhibit a notable decay in current over time for all measurement configurations, but much less for the lower temperatures.

**Figure 3:**
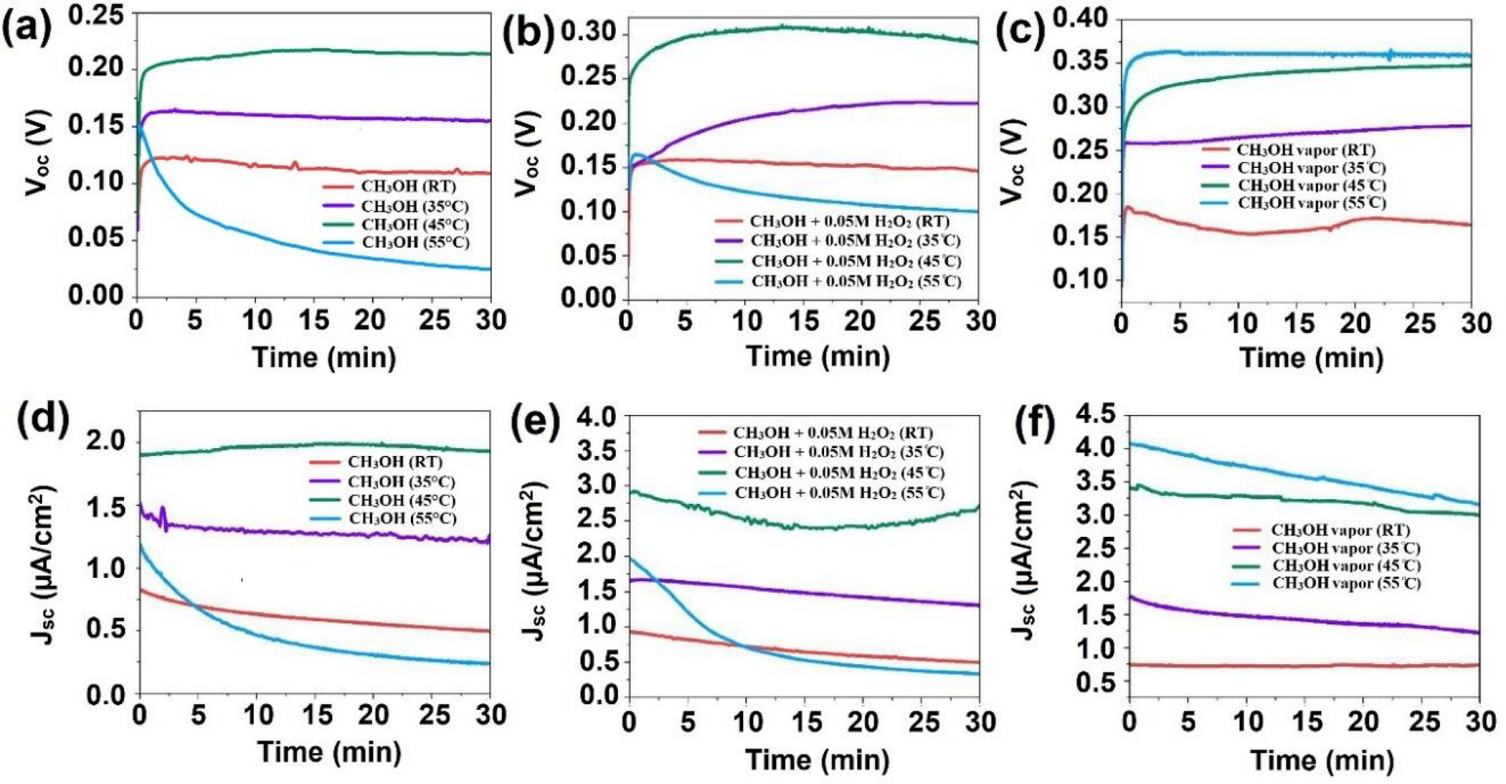
Chronopotentiometry curves of the GaAs/GaInP cell in the presence of CH_3_OH (a), CH_3_OH+(0.05 M) H_2_O_2_ (b) solutions and CH_3_OH vapor (c). Corresponding Chronoamperometry curves in the presence of CH_3_OH (d), CH_3_OH+(0.05 M) H_2_O_2_ (e) solutions and CH_3_OH vapor (f).

To illustrate the fundamental aspects of the chemovoltaic effect, Figure 4 presents the band diagram for a GaAs/GaInP double heterojunction and the energy levels of the methanol + oxygen system. In steady-state conditions, the conduction band quasi-Fermi level (*E*_fc_) of GaAs resides below the electrode potential necessary for the methanol oxidation reaction, enabling electron injection from the methanol oxidation reaction into the conduction band (CB) of GaAs.^5^ Similarly, the oxygen reduction reaction provides holes to the valence band (VB) of GaAs, given that the valence band quasi-Fermi level (*E*_fv_) of GaAs is higher than the electrode potential of the O_2_ reduction reaction.^5^ As shown in Figure 4, the chemically injected electrons in the CB of p-GaAs will tend toward the CB of n-GaInP due to diffusion and the p–n junction. The injected electrons are finally transferred to the n contact (AuGe/Ni) and then to the p contact (Au/Ti) through an external circuit, whereas the injected holes are transferred to the p-type contact through the p-GaInP (shown by the equivalent arrow in Figure 4) and p^++^-GaAs layers.

**Figure 4:**
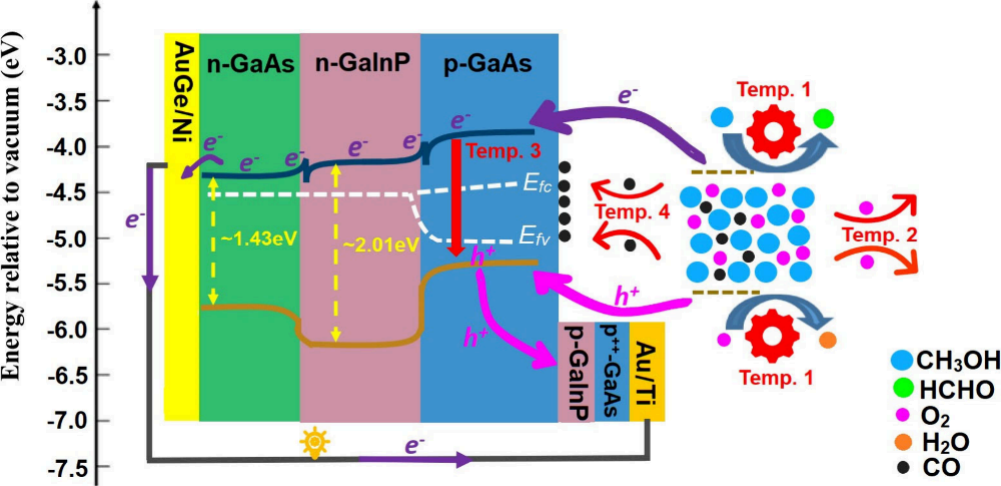
Schematic band diagram for a GaAs/GaInP heterojunction exposed to a CH_3_OH + O_2_ solution. The diagram also shows indicative band alignments for methanol oxidation and oxygen reduction potentials at standard conditions and illustrates various competing mechanisms through which the temperature can affect the performance, including the accelerated rates of redox reactions (Temp. 1), escape of dissolved O_2_ molecules from the semiconductor surface (Temp. 2), nonradiative recombination of the injected charge carriers (Temp. 3), and the poisoning effect as a result of CO absorption on the surface (Temp. 4).

The charge transfer kinetics might also be affected by band bending at the p-GaAs surface. The three different possibilities for the surface band bending (i.e., upward, flat-band, and downward) at the p-GaAs surface and the corresponding charge carrier injections are shown in Figure S3. The upward (Figure S3a) or downward (Figure S3b) band bending shifts the positions of the quasi-Fermi levels for electrons (*E*_fc_) and holes (*E*_fv_), thereby altering the driving force for electron and hole injection into the conduction and valence bands with respect to the corresponding flat-band condition. However, the effect of band bending on overall cell performance is likely affected by several different mechanisms, similar to the case of photocatalytic water splitting^10^, and warrants further study.

For additional discussion on the several possibly competing effects in the measurements, Figure 4 also maps out selected potential impacts of temperature on the charge transfer process. The *J*–*V* results of liquid- and vapor-phase measurements suggest that elevated temperatures up to a certain threshold (*T* = 45 °C in this study) positively influence the methanol oxidation and/or the oxygen reduction reactions, and the respective reaction barriers, labeled as mechanism “Temp. 1” in Figure 4. This enhancement is associated with increased electron (hole) injection into the conduction (valence) band of the semiconductor, resulting in better performance of the chemovoltaic cell. However, a further increase in the temperature of the liquid system (e.g., *T* = 55 °C) results in degraded cell performance, likely due to the escape of O_2_ molecules from the solution, decreasing the oxygen concentration through the mechanism “Temp. 2” in Figure 4. As a result, there is an insufficient oxygen concentration in the vicinity of the semiconductor surface, resulting in a diminished rate of hole injection into the valence band. Consequently, this hinders the chemovoltaic effect on the semiconductor surface, which relies on electron and hole injections to the conduction and valence bands of the semiconductor by simultaneous fuel oxidation and oxidant reduction reactions, respectively. However, for the vapor-phase measurements, where the availability of oxygen molecules is not expected to change significantly even close to 55 °C, the cell *V*_oc_ and *J*_sc_ increase beyond the corresponding maxima of the liquid-phase measurements.

In addition to reagent availability, the performance may be affected by the increased temperature on the chemovoltaic cell itself, as higher temperatures tend to increase the nonradiative recombination rates within the cell, as indicated by the mechanism “Temp. 3”. Similarly, there is another competing current that is generated as a result of the injection of carriers by an external bias. When the external bias exceeds the value of *V*_oc_, the recombination of the charge carriers in the depletion region of the p–n junction exceeds the generation rate of the chemical excitation.

During the exposure to the chemicals/vapors, the GaAs surface is likely altered chemically as well and can be, e.g., oxidized or poisoned, as indicated by mechanism “Temp. 4”. Such chemical changes might be the reason for the slight current decay observed for both liquid and vapor systems at almost all temperatures (see Figure 3e,f), although *V*_oc_ remains nearly constant throughout the measurement time (see Figure 3a–c) except for the liquid-phase measurement at 55 °C.

It is obvious that the semiconductor surface plays a vital role in the performance of the SEFC devices based on the associated chemovoltaic effect. This opens possibilities to engineer the semiconductor surface by suitable catalysts, which could directly influence the current densities by increasing the reaction rates at the electrodes, thereby enhancing the overall efficiency and performance of the fuel cell.

The catalysts’ functions also extend beyond reducing the charge transfer resistance and activation energy associated with methanol oxidation, as a suitable catalyst provides more tolerance to poisoning by methanol-related species such as CO_ads_.^6,11−14^ For example, Mahapatra et al.^6^ reported that using Pt–Pd/C electrocatalysts in an alkaline medium of direct methanol fuel cells (DMFCs) results in facile adsorption of OH^–^ on the Pt surface, which reacts with the carbon monoxide molecules, facilitating the methanol oxidation reaction without serious poisoning effects. More recently, anti-CO poisoning ability of ternary FePtRh nanoflowers has been reported by Liu and co-workers.^11^ This is due to efficient electron transfer from Pt to Fe or Rh atoms, which weakens the strong adsorption energy between poisoning CO and Pt atoms. Hence, surface engineering of the chemovoltaic semiconductor device with optimized catalysts could potentially result in substantial enhancements in electricity generation by providing a nonpoisoning surface. This avenue remains open for further exploration in follow-up studies.

Overall, the highest *J*_sc_ obtained in this work in the presence of liquid and vapor methanol fuels is over one order of magnitude higher than those reported in our previous work,^5^ and the highest *V*_oc_ is twice as high. This marks a significant step toward understanding the kinetics of the energy conversion process for a promising electricity generation procedure that can convert solar energy into electricity using the photovoltaic effect during the daytime and simultaneously produce power via the chemovoltaic effect of fuel cells during the nighttime.

In summary, we have demonstrated an enhanced chemovoltaic effect utilizing a double heterojunction diode in the presence of both liquid- and vapor-phase methanol, examining its temperature dependence. The experimental results showed that increasing the temperature to an optimal value (ca. 45 °C for the liquid phase in this work) significantly improves the SEFC performance for both liquid and vapor methanol fuels due to higher rates of the oxidation and reduction reactions which inject electrons and holes to the conduction and valence bands of the GaAs cell, respectively. Our findings provide a significant advance in understanding the energy conversion kinetics for generating electricity through the chemovoltaic effect, which allows developing hybrid solar–fuel cells that can overcome geometry- and electrolyte-related limitations of conventional fuel cells. These findings can also introduce a new research topic in the field of surface sciences and pave the way for new opportunities in the development of sustainable energy conversion devices and chemical detectors.

## Supporting Information Available

*J*–*V* curves and schematic illustration of band diagrams (PDF)

## References

[ref1] Grove W. R. XXIV. On voltaic series and the combination of gases by platinum. London, Edinburgh Dublin Philos. Mag. J. Sci. 1839, 14 (86–87), 127–130. 10.1080/14786443908649684.

[ref2] Steele B. C. Material science and engineering: the enabling technology for the commercialisation of fuel cell systems. J. Mater. Sci. 2001, 36, 1053–1068. 10.1023/A:1004853019349.

[ref3] Steele B. C.; Heinzel A. Materials for fuel-cell technologies. Nature 2001, 414 (6861), 345–352. 10.1038/35104620. 11713541

[ref4] Wilson J. R.; Kobsiriphat W.; Mendoza R.; Chen H.-Y.; Hiller J. M.; Miller D. J.; Thornton K.; Voorhees P. W.; Adler S. B.; Barnett S. A. Three-dimensional reconstruction of a solid-oxide fuel-cell anode. Nat. Mater. 2006, 5 (7), 541–544. 10.1038/nmat1668. 16767095

[ref5] Alizadeh M.; Radevici I.; Li S.; Oksanen J. Chemovoltaic effect for renewable liquid and vapor fuels on semiconductor surfaces. ChemSusChem 2024, 17 (5), e202301522 10.1002/cssc.202301522. 38305144

[ref6] Mahapatra S.; Dutta A.; Datta J. Temperature dependence on methanol oxidation and product formation on Pt and Pd modified Pt electrodes in alkaline medium. Int. J. Hydrogen Energy 2011, 36 (22), 14873–14883. 10.1016/j.ijhydene.2010.11.085.

[ref7] Paredes-Salazar E. A.; Calderón-Cárdenas A.; Herrero E.; Varela H. Unraveling the impact of temperature on the reaction kinetics of the electro-oxidation of methanol on Pt (100). J. Catal. 2024, 432, 115402 10.1016/j.jcat.2024.115402.

[ref8] Gawel L.; Parasinska D. Dynamic impedance measurements of the Direct Methanol Fuel Cell cathode at various operating temperatures. Int. J. Hydrogen Energy 2024, 67, 83–90. 10.1016/j.ijhydene.2024.04.169.

[ref9] Li S.; Chen K.; Alizadeh M.; Vähänissi V.; Savin H.; Oksanen J. Adsorption induced bipolar excitation at semiconductor surface. Surf. Interfaces 2024, 50, 104499 10.1016/j.surfin.2024.104499.

[ref10] Kaufman A. J.; Nielander A. C.; Meyer G. J.; Maldonado S.; Ardo S.; Boettcher S. W. Absolute band-edge energies are over-emphasized in the design of photoelectrochemical materials. Nat. Catal. 2024, 7 (6), 615–623. 10.1038/s41929-024-01161-0.

[ref11] Liu H.; Jia R.; Qin C.; Yang Q.; Tang Z.; Li M.; Ma Z. Anti-CO Poisoning FePtRh Nanoflowers with Rh-Rich Core and Fe-Rich Shell Boost Methanol Oxidation Electrocatalysis. Adv. Funct. Mater. 2023, 33 (7), 2210626 10.1002/adfm.202210626.

[ref12] Chung D. Y.; Kim H.-i.; Chung Y.-H.; Lee M. J.; Yoo S. J.; Bokare A. D.; Choi W.; Sung Y.-E. Inhibition of CO poisoning on Pt catalyst coupled with the reduction of toxic hexavalent chromium in a dual-functional fuel cell. Sci. Rep. 2014, 4 (1), 7450 10.1038/srep07450. 25502744 PMC4264001

[ref13] Chen X.; Granda-Marulanda L. P.; McCrum I. T.; Koper M. T. How palladium inhibits CO poisoning during electrocatalytic formic acid oxidation and carbon dioxide reduction. Nat. Commun. 2022, 13 (1), 38 10.1038/s41467-021-27793-5. 35013444 PMC8748733

[ref14] Hsieh Y.-C.; Zhang Y.; Su D.; Volkov V.; Si R.; Wu L.; Zhu Y.; An W.; Liu P.; He P.; Ye S.; Adzic R. R.; Wang J. X Ordered bilayer ruthenium–platinum core-shell nanoparticles as carbon monoxide-tolerant fuel cell catalysts. Nat. Commun. 2013, 4 (1), 2466 10.1038/ncomms3466. 24045405

