# Supplementary material for: Temperature Dependence of Reaction Kinetics in a Hybrid GaAs Solar–Fuel Cell Device
Source: J Phys Chem Lett. 2024 Sep 10;15(37):9487–92. doi: 10.1021/acs.jpclett.4c02018 (PMC11498716; doi:10.1021/acs.jpclett.4c02018)
Supplement: Supplementary file 1 — jz4c02018_si_001.pdf [file jz4c02018_si_001.pdf]

Supplementary materials

Temperature Dependence of Reaction Kinetics in a Hybrid GaAs Solar-Fuel Cell Device

Mahdi Alizadeh[1], Shengyang Li[2], Seyed Ahmad Shahahmadi[1], Jani Oksanen[1]*

[1]Engineered Nanosystems Group, School of Science, Aalto University, Tietotie 1, Espoo, 02150, Finland

[2]Department of Chemistry, China Agricultural University, Beijing 100193, China

[*]Corresponding author: mahdi.alizadehkouzehrash@aalto.fi

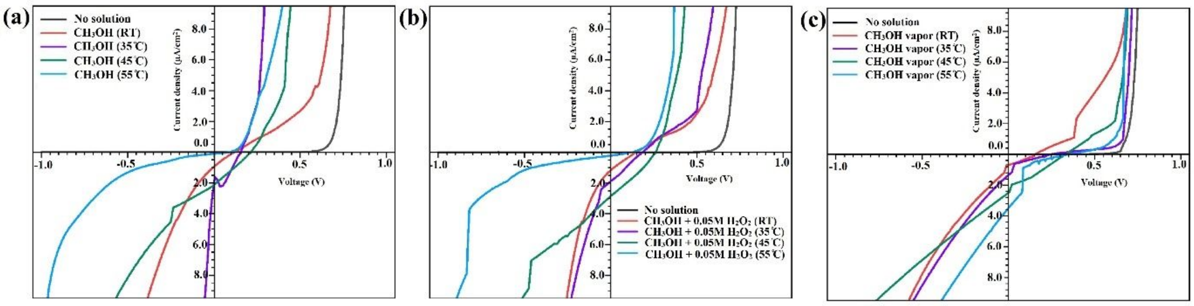

Figure S1: Linear J–V curves of the GaAs/GaInP cell in the dark under different temperatures with and without exposure to CH_3OH (a), CH_3OH+(0.05 M) H_2O_2 (b) and CH_3OH vapor (c).

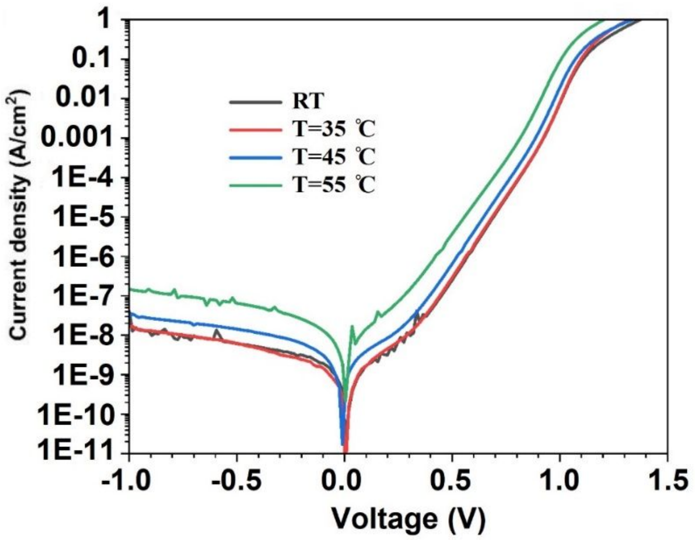

Figure S2: J–V curves of the GaAs/GaInP cell in the dark under different temperatures and without exposure to solution.

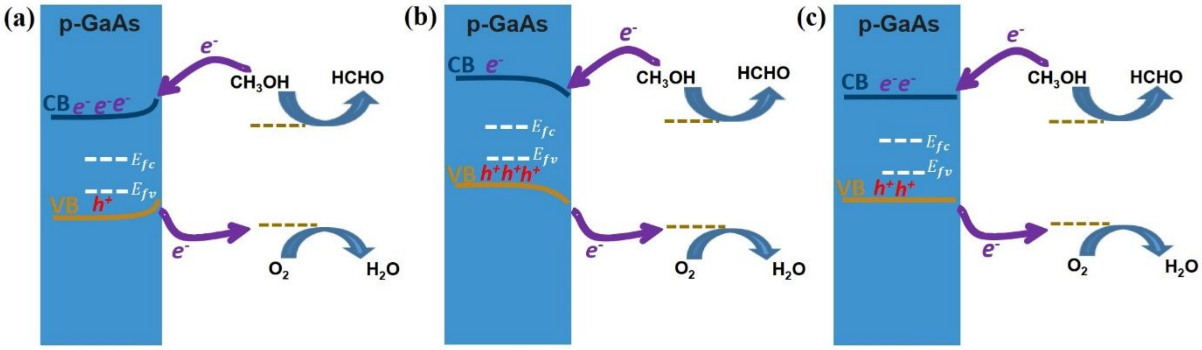

Figure S3: Schematic illustration of the band diagrams for upward band bending (a), downward band bending (b) and flat band conditions (c) and corresponding charge injections from the redox reactions.
